# Supplementary material for: Sharp symmetry-change marks the mechanical failure transition of glasses
Source: Sci Rep. 2015 Sep 25;5:14359. doi: 10.1038/srep14359 (PMC4585902; doi:10.1038/srep14359)
Supplement: Supplementary Information [file srep14359-s1.pdf]

Supporting Information for article "Sharp symmetry-change marks the mechanical failure transition of glasses"

D. V. Denisov,[1] M. T. Dang,[1] B. Struth,[2] A. Zaccone,[3] G. H. Wegdam,[1] and P. Schall[1]

[1] *Van der Waals-Zeeman Institute, University of Amsterdam, The Netherlands*

[2] *Deutsches Elektronen-Synchrotron, Hamburg, Germany*

[3] *Physics Department and Institute for Advanced Study, Technische Universität München, 85748 Garching, Germany*

I. ELASTIC CAGE DISTORTION AND THE ORIGIN OF P-WAVE SYMMETRY

In this section, we show that under an affine cage distortion, the structure factor in the imaged plane (shear direction - shear axis plane) acquires the p-wave symmetry observed in the experiments at small strain. Let x be the direction of shear, z the shear gradient direction, and y the shear axis as shown in Fig. 1c of the manuscript. When the cage is distorted according to the applied shear field, there is an important component of affine deformation as long as the system reacts elastically (i.e. solid-like). The affine displacements due to the applied shear tend to distort the cage in the $x - z$ plane as shown in Fig. 1c. In addition to the affine displacements, at a non-zero shear rate, there are also displacements along the vorticity lines (the red arrows in that figure) of the shear field: Particles tend to be depleted in the extension sectors of the $x - z$ plane, and they tend to accumulate and crowd in the compression sectors. The combined effects of vorticity, crowding, and excluded-volume (hard-sphere repulsion) between particles in the compression sectors lead to a force dipole along the x-direction. As shown in Fig. 1c in the main article, the center of mass of the dipole coincides with the tagged particle at the center of the cage.

The elastic field of the force dipole and its component in the imaged $x - y$ plane can be calculated as follows. The elastic field obeys the Poisson-like equation $K\nabla^2\mathbf{u} = -\mathbf{f}(\mathbf{r})$, where K is the bulk modulus, and $\mathbf{f}$ is the local force dipole given by $\mathbf{f}(\mathbf{r}) = f_0[\epsilon \cdot \nabla\delta(\mathbf{r})]\mathbf{n}$. The dipole moment is given by $\mathbf{p} = f_0\epsilon$, with $\epsilon = \epsilon\mathbf{n}$, and the Dirac delta is centred at the center of mass of the dipole, i.e. on the tagged particle at the center of the cage which we take as the origin of the spherical reference frame ($\mathbf{r} = 0$). The solution to the Poisson equation for the elastic field induced by the force dipole is given in analogy with the electrostatic problem (field induced by an electric dipole), and is given by [1]

$$\mathbf{u}(\mathbf{r}) = -\frac{p}{4\pi K}\frac{\mathbf{r}\cdot\mathbf{n}}{r^3}\mathbf{n}. \quad (1)$$

In the spherical frame, the position vector is defined as $\mathbf{r} = (r_x, r_y, r_z) = (r\sin\theta\cos\varphi, r\sin\theta\sin\varphi, r\cos\theta)$. Similarly, the unit vector defining the dipole direction $\mathbf{n} = (n_x, 0, 0_z) = (\sin\theta\cos\varphi, 0, 0)$. Here, θ is the polar angle, measured with respect to the z-axis, while ϕ is the azimuthal angle in the $x - y$ plane, measured with respect to the $x-$direction. Therefore $\phi \equiv \alpha$. Upon replacing the expressions for $\mathbf{r}$ and $\mathbf{n}$ in polar coordinates in Eq.(1) above, we immediately get the angular dependence of the magnitude of elastic displacement in the $x - y$ plane by choosing $\theta = \pi/2$. We obtain the following angular dependence of the elastic field in the $x - y$ plane

$$u(\alpha) \propto \mathbf{r}\cdot\mathbf{n} \propto \sin^2\theta\cos^2\alpha = \cos^2\alpha \quad (2)$$

where in the last equality we used $\sin^2\theta = 1$ for $\theta = \pi/2$ ($x - y$ plane). This derivation demonstrates that the force dipole along the $x-$direction caused by the affine cage distortion in Fig. 1c induces an elastic field in the $x - y$ plane which goes as $\cos^2\alpha$, i.e. has p-wave symmetry. This is indeed the symmetry of the structure factor observed experimentally for the sheared glass.

II. FLUCTUATIONS AND INTER CYCLE STRUCTURE

We independently confirmed the sharp dynamically-induced transition in the fluctuations of the structure factor. To do so, we investigated in detail fluctuations and their time correlation during the oscillatory shear. Recent work on the oscillatory rheology of soft glasses suggests that the material may exhibit a succession of straining, yielding, and flow within each period of oscillation [2]. We thus investigated, in our x-ray data, the presence of any typical time scale of yielding and flow within the underlying oscillation period. We used both time correlation and Fourier analysis to look for such typical time scale. We compute time correlations by correlating order parameter values at times t and $t + \Delta t$ using

$$F(\Delta t) = \frac{1}{T}\int_0^T (C(t+\Delta t) - < C(t) >)(C(t) - < C(t) >)dt, \quad (3)$$

where $t \sim \log(\gamma_0/\gamma_{0min})$ and we correlate order parameter values $C(t) = C(\beta = \pi, t)$ as a function of delay time $\Delta t \sim \Delta\log(\gamma_0)$. Here, T is the averaging time interval. A typical result obtained for sufficiently large averaging time interval is shown in Fig. Ia. No characteristic time scale is observed. The data rather suggests that the fluctuations are random and due to noise. This is confirmed

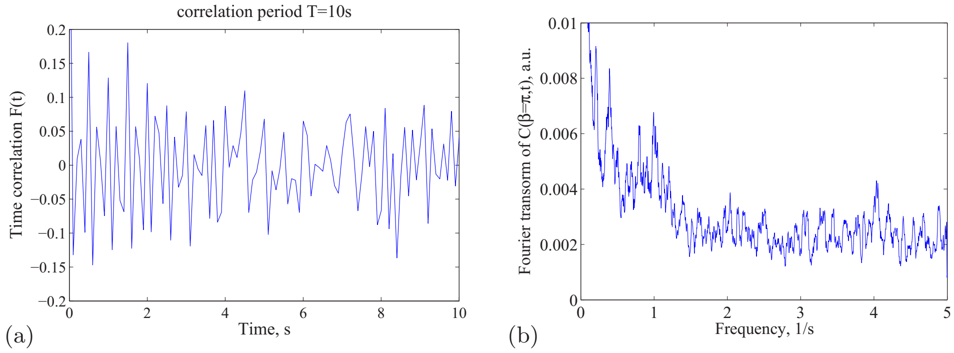

FIG. I. **Time correlation and Fourier analysis of structure factor.** (a) Autocorrelation function $F(t)$ of the fluctuations of the order parameter $C(\beta = \pi, 0)$ in the non-linear regime after symmetry-breaking. Averaging was done over $T = 10$ seconds corresponding to 100 frames. (b) Fourier analysis of the same quantity. Only a very weak peak is found at the characteristic frequency 1 Hz.

independently by Fourier analysis: we fourier-transform the same signal and show the Fourier amplitude as a function of frequency in Fig. Ib. Although the data might suggest that there is a weak peak at 1Hz (and possibly 0.5 Hz), its amplitude is of the order of the noise. We hence cannot unambigously detect a typical frequency related to the applied shear oscillation, and we interpret the fluctuations as noise . Further work with higher time resolution is needed to detail this point. Because of the lack of a characteristic time scale, for the order parameter in the manuscript, we average over one oscillation period, i.e. we compute time averages using eq. 3 with $\Delta t = 1s$, the oscillation period.

III. GLASS-LIQUID TRANSITION CRITERION

How can we understand, from a microscopic viewpoint that the transition from solid to liquid-like response occurs exactly at the intersection of the moduli, $G' = G''$? On a quantitative level, it can be explained as follows: The viscoelastic response of the amorphous solid can be described schematically as composed of three contributions: the affine elastic modulus G_A, the negative nonaffine contribution $-G_{NA}$ due to structural disorder, and the contribution due to relaxation, $G_R\ e^{-t/\tau_R}$, where τ_R is the relaxation time due to internal friction. Hence, $G = G_A - G_{NA} + G_R\ e^{-t/\tau_R}$ [3, 4], with $G_A - G_{NA} \equiv G_\infty$ being the infinite-time, elastic part of the response. One should note that G_A is related to local bonding and may change with the applied strain γ due to irreversible changes in the local connectivity. Upon Fourier-transforming we obtain the standard form $G' = G_R + (G_\infty - G_R)[1 + (\omega\tau_R)^2]^{-1}$ [4]. Previous work based on the non-affine response formalism [3, 5] has shown that the elastic response of an athermal amorphous solid vanishes when the nonaffine contribution becomes equal to the affine contribution, which is the point at which rigidity vanishes: $G_\infty = G_A - G_{NA} = 0$. At this point, we necessarily have $G' = G_R(1 - [1 + (\omega\tau_R)^2]^{-1}) = G_R(\omega\tau_R)^2/[1 + (\omega\tau_R)^2]$. Next, recall the form of the loss modulus in the same standard linear solid model [4] as $G'' = G_R\omega\tau_R/[1 + (\omega\tau_R)^2]$. At intermediate frequencies which probe the material's viscoelastic response, $\omega\tau_R \simeq 1$, therefore, the condition $G_\infty = G_A - G_{NA} = 0$ leads straight to the equality $G' \simeq G''$. This equality must hold, at least approximately, at the rigidity transition of an amorphous solid, where $G_\infty = 0$ due to nonaffine motions becoming overwhelming with respect to the affine motions [5]. This transition in the viscoelastic regime (finite or intermediate frequency) cannot be very sharp because it is partly obscured by the relaxational contribution to the modulus ($\propto G_R$) which remains finite even though the zero-frequency/infinite-time part (G_∞) has vanished at the rigidity transition. This argument provides an additional indication that it is indeed the transition from the affine-dominated regime $G_A >| G_{NA} |$ (associated with the shear-induced symmetry) into the nonaffine-dominated regime $G_A <| G_{NA} |$ (associated locally with disorder and fluctuations) that we probe in our experiment. We hence show experimentally that indeed the affine to non-affine rigidity transition is *the* key microscopic phenomenon controlling the macroscopic criterion $G' = G''$ at the onset of flow. The fact that we recover $G' = G''$ as a criterion for the transition from a solid to a liquid state from our microscopic mechanism proofs the robustness of our proposed scenario.

[1] Landau LD, Lifshitz EM (1960) *Electrodynamics of Continuous Media* (Pergamon Press, Oxford).

[2] Rogers SA, Erwin BM, Vlassopoulos D, Cloitre M (2011) Oscillatory yielding of a colloidal star glass. *J. Rheol.*

55(4):733-752.
[3] Lemaître A, Maloney C (2006) Sum Rules for the Quasi-Static and Visco-Elastic Response of Disordered Solids at Zero Temperature. *J. Stat. Phys.* 123(2):415-453.
[4] Zener C (1948) *Elasticity and anelasticity of metals* (Chicago Univ. Press, Chicago).
[5] Zaccone A, Scossa-Romano E (2011) Approximate analytical description of the nonaffine response of amorphous solids. *Phys. Rev. B* 83(18):184205.
